# Impact of a Web-Based Exercise and Nutritional Education Intervention in Patients Who Are Obese With Hypertension: Randomized Wait-List Controlled Trial

**DOI:** 10.2196/14196

**Published:** 2020-04-14

**Authors:** Juan Francisco Lisón, Gonzalo Palomar, Marinna S Mensorio, Rosa M Baños, Ausiàs Cebolla-Martí, Cristina Botella, Vicent Benavent-Caballer, Enrique Rodilla

**Affiliations:** 1 Department of Medicine Universidad Cardenal Herrera CEU, CEU Universities Valencia Spain; 2 Centro de Investigación Biomédica en Red Fisiopatología Obesidad y Nutrición Instituto Carlos III Madrid Spain; 3 Primary Care Health Center Quartell Spain; 4 Coordenação de Aperfeiçoamento de Pessoal de Nível Superior Foundation Brasilia Brazil; 5 Department of Personality, Assessment and Psychological Treatment Facultad de Psicología Universitat de València Valencia Spain; 6 Universitat Jaume I Castellón Spain; 7 Department of Physiotherapy Universidad Cardenal Herrera CEU, CEU Universities Valencia Spain; 8 Hypertension and Vascular Risk Unit Hospital de Sagunto Sagunto Spain

**Keywords:** web, internet, overweight, obesity, hypertension

## Abstract

**Background:**

Internet-based interventions are a promising strategy for promoting healthy lifestyle behaviors. These have a tremendous potential for delivering electronic health interventions in scalable and cost-effective ways. There is strong evidence that the use of these programs can lead to weight loss and can lower patients’ average blood pressure (BP) levels. So far, few studies have investigated the effects of internet-based programs on patients who are obese with hypertension (HTN).

**Objective:**

The aim of this study is to investigate the short- and long-term efficacy, in terms of body composition and BP parameters, of a self-administered internet-based intervention involving different modules and learning techniques aimed at promoting lifestyle changes (both physical activity and healthy eating) in patients who are obese with HTN.

**Methods:**

A randomized wait-list controlled trial design was used. We recruited 105 adults with HTN who were overweight or obese and randomly assigned them to either a 3-month internet-based intervention group (n=55) or the wait-list control group (n=50). We assessed BMI (primary outcome), body fat mass (BFM), systolic (S)BP and diastolic (D)BP, blood glucose and insulin levels, physical activity levels, and functional capacity for aerobic exercise at Time 0 (preintervention) and Time 1 (postintervention). All the patients in the wait-list control group subsequently received the intervention, and a secondary within-group analysis, which also included these participants, was conducted at Time 2 (12-month follow-up).

**Results:**

A 2-way mixed analysis of covariance showed a significant decrease in BMI, BFM, and blood glucose at 3 months in the internet-based intervention group; the effect size for the BMI and BFM parameters was moderate to large, and there was also a borderline significant trend for DBP and insulin. These results were either maintained or improved upon at Time 2 and showed significant changes for BMI (mean difference −0.4, 95% CI −0.1 to −0.6; *P*=.005), BFM (mean difference −2.4, 95% CI −1.1 to −3.6; *P*<.001), DBP (mean difference −1.8, 95% CI −0.2 to −3.3; *P*=.03), and blood glucose (mean difference −2, 95% CI 0 to −4; *P*=.04).

**Conclusions:**

Implementation of our self-administered internet-based intervention, which involved different learning techniques aimed to promote lifestyle changes, resulted in positive short- and long-term health benefits in patients who are obese with HTN.

**Trial Registration:**

ClinicalTrials.gov NCT03396302; https://clinicaltrials.gov/ct2/show/NCT03396302

## Introduction

### Background

Cardiovascular disease is the leading cause of morbidity and mortality in developed countries and continues to be a major public health issue that accounts for over 4 million deaths per year in Europe [1]. Obesity, which has been described as a global pandemic [2], is one of the most significant medical threats. It is associated with early death [3,4] and is universally recognized as a risk factor for many health complications such as hypertension (HTN). Because a strong association between blood pressure (BP) and body weight has previously been documented [5,6], the increasing prevalence of HTN is thought to be linked to the dramatic increase of individuals who are overweight or obese [7]. As the prevalence, health consequences, and costs of obesity and HTN rise, clinicians and researchers are continuing to investigate a variety of treatment options for these patients.

Promoting healthy behaviors such as physical activity and healthy eating through lifestyle counselling is recommended as the first-line therapy for the treatment of these patients and may be an effective tool for treating obesity and preventing obesity-related health burdens [6,8,9]. However, frequent visits to outpatient clinics are costly and time-consuming for patients as well as for physicians and nurse practitioners. These patients need to develop specific self-management skills so that they can make long-lasting lifestyle changes and adhere to their treatments; however, clinics may not be an ideal environment for them because these surroundings can be perceived as intrusive and threatening [10,11]. Furthermore, in many cases physicians and nurses do not have the proper background training to provide patients with the best possible counselling about nutrition or physical activity. Therefore, an approach is needed that can be offered without imposing additional burdens on our health care workers or budget.

Internet-based interventions (IBIs) are a promising strategy for promoting healthy lifestyle behaviors and have not been entirely explored; these have a tremendous potential for delivering electronic health (eHealth) interventions in scalable and cost-effective ways. IBIs can provide immediate, easy to access, relatively inexpensive, and individually tailored support for self-management and the promotion of behavioral change to large segments of the population. Moreover, there is strong evidence that the use of these programs can lead to weight loss [12-21] and can lower average patient BP levels [21-26]. So far, few studies have investigated the effects of internet-based programs on patients who are obese with HTN [27-29]. Furthermore, to the best of our knowledge, no study has assessed the efficacy of a self-administered IBI, which entails the completion of different modules and incorporates several learning techniques aimed at promoting lifestyle changes in patients who are obese with HTN.

### Aim and Hypotheses

This study aimed to examine the short- and long-term efficacy in terms of body composition and BP parameters of a self-administered IBI involving different modules and learning techniques, aimed at promoting lifestyle changes (both physical activity and healthy eating) in patients who are obese with HTN.

Compared with the wait-list control (WLC) group receiving standard medical care, we hypothesized that the IBI group will have significantly better improvements for body composition, BP, blood glucose and insulin levels, physical activity levels, and functional capacity for aerobic exercise from baseline to postintervention.

## Methods

### Study Design

This prospective, single-center, wait-list controlled trial (trial registration: ClinicalTrials.gov NCT03396302) with balanced randomization (1:1) was approved by the Hospital of Sagunto Human Ethics Committee and followed the ethical guidelines set out in the Declaration of Helsinki.

### Eligibility Criteria

Eligible participants were all adults between 18 and 65 years of age with HTN and who were overweight (BMI>24.9 kg/m^2^ and <30 kg/m^2^) or had type 1 obesity (BMI>29.9 kg/m^2^ and <35 kg/m^2^). HTN was defined as systolic (S)BP≥140 mmHg or diastolic (D)BP≥90 mmHg, or current use of antihypertensive medication. All patients in our study were on antihypertensive treatment. The exclusion criteria included a diagnosis of diabetes, previous ischemic heart disease, cerebrovascular disease, or a severe psychiatric disorder; taking more than three antihypertensive drugs; physical impairments precluding participation in physical activity; receiving any treatment for weight loss elsewhere; or having no internet access.

### Procedure

**Figure 1:**
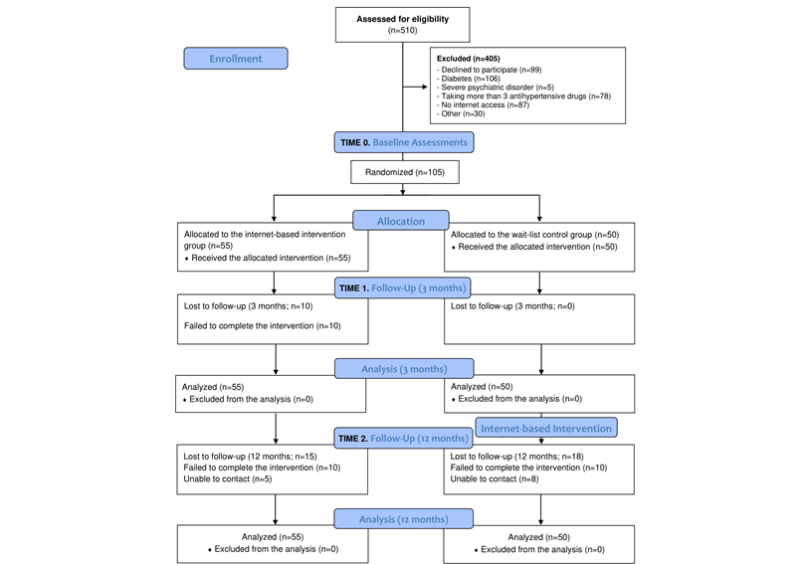
Progression of the participants through the trial.

This study took place at the HTN and Vascular-Risk Unit at the hospital of Sagunto (Spain), from January 2018 to March 2019. The study included 105 participants. Before the start of the trial, Researcher 1, who was not involved in the recruitment or inclusion of the participants, generated a random sequence (based on simple randomization) using a computerized random number generator; this was concealed from all other study investigators throughout the entire study period. Upon enrollment in the study and after completing the primary and secondary outcome measures (baseline), the participants were randomly assigned either to the 3-month IBI group (n=55) or the WLC group (n=50). As shown in the participant flowchart in Figure 1, all the outcome measures were assessed at baseline (Time 0), 3 months postbaseline (Time 1), and at a 12-month follow-up (Time 2) for the IBI group. The WLC group took part in an initial baseline assessment (Time 0) followed by a second assessment 3 months postbaseline (Time 1).

**Figure 2:**
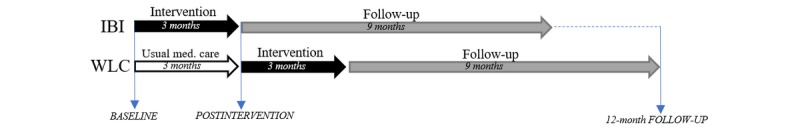
Measurements at trial profile. IBI: internet-based intervention group; WLC: wait-list control group.

After the Time 1 assessment, WLC participants received the intervention, which was offered to comply with the instructions of the hospital’s human ethics committee, and the patients were subsequently assessed at a 12-month follow-up (Time 2) (Figure 2). Thus, all the participants underwent a preintervention, postintervention (3 months), and a 12-month follow-up assessment, and the WLC participants were assessed twice before the intervention. All the outcome measurements were recorded in both groups by two trained researchers who were blinded to the group allocation.

### Intervention

The intervention program we used is called “Vivir mejor” (translated from Spanish as “Live better”) [27,30]. For this intervention, in addition to usual medical care, participants received a 3-month multimedia, interactive, and self-administered online intervention program comprised of nine modules (Multimedia Appendix 1). These modules specifically focus on obesity and HTN, and are presented via a webpage that aims to progressively establish healthy eating habits and increase the patient’s physical activity levels, as recommended by the World Health Organization’s guidelines [31,32]. The first 5 modules were activated at a rate of one per week, and modules 6 to 9 were activated every 2 weeks; thus, the intervention lasted for 3 months. The program was delivered in the participants’ native language (Spanish) and included psychoeducation about what a healthy lifestyle involves and taught techniques for how this can be achieved on a day-to-day basis. Some of the techniques used were self-monitoring, self-instruction, behavioral recording, stimulus control, self-reinforcement, problem-solving techniques, and homework. In addition, the webpage offered useful tools such as downloadable documents and videos. This program is described in more detail in Baños et al [30].

During the first 3 months of the study and prior to starting the intervention, the WLC group received standard medical care that focused on reducing their cardiovascular risk factors [33]. Standard medical care included antihypertensive prescription, written lifestyle advice, moderate salt restriction with a list of recommended foods, a low-calorie diet, and advice for physical activity, which aimed for 5 sessions per week and a recommended maximum heart rate during exercise adjusted to age. The usual care program did not provide any classes for the subjects. Standard medical care was carried out exclusively by the public National Health System, which the Hypertension Unit belongs to.

This IBI was delivered using a web-based platform called Wix. Wix is a self-hosted website builder and content management system with more than 90 million users [34]. This cloud-based website development platform is customizable with drag-and-drop features and includes apps, graphics, image galleries, fonts, and a responsive design that adjusts the site for viewing with mobile devices. We purchased a unique website domain URL to avoid pop-up advertising from Wix. To prevent the general public from accessing the site, a password was added to secure the proprietary information and participant responses [35].

### Outcome Measures

BMI and secondary outcomes (body fat mass [BFM], SBP and DBP, plasma glucose, insulin, habitual level of physical activity, and functional capacity for aerobic exercise) were measured using validated and reliable tests. BMI (kg/m^2^) was calculated using an electronic balance scale (SECA 780 with a mechanical telescopic stadiometer).

BFM was determined using a body-fat analyzer (Tanita SC-331S). SBP and DBP were measured with a validated, semi-automatic, digital tensiometer (Colin Press-Mate BP-8800) according to the European Society of Hypertension and European Society of Cardiology guidelines, as described elsewhere [36,37]. All the assessments (baseline, 3 month postintervention, and 12-month follow-up) were scheduled between 8 am and noon to minimize diurnal BP variability. Blood samples were obtained in the morning after a minimum 8 hours of fasting. Serum biochemical profiles were measured using a multiple-channel autoanalyzer. Plasma glucose was assayed using the glucose oxidase method (Beckman Glucose Analyzer).

At Time 0, the patients’ habitual levels of physical activity were objectively monitored for 7 consecutive days using an accelerometer (Actigraph GT3X) [36]. The participants were carefully instructed on how to attach the activity monitor (using an elastic belt), which was to be worn each day for the whole measurement period, and were asked to wear the monitor during the daytime; exceptions were made during the performance of water activities such as swimming or bathing. The total amount of physical activity recorded by the accelerometer was expressed as the average of the total counts per minute of the registered time.

A submaximal exercise test, the 6-minute walk test (6MWT), was used to assess the participants’ functional capacity for aerobic exercise. This test evaluates the maximum distance that they could cover along a 30-m long corridor during a 6-minute period. Participants were instructed to walk along the walkway as fast as possible and to stop when needed. The assessor walked alongside the participants to ensure their safety and provided them with standardized verbal encouragement at 1, 3, and 5 minutes, and the total distance covered (in meters) was recorded. Participants were instructed to avoid smoking for 48 hours, caffeine for 12 hours, and strenuous exercise for 24 hours prior to their assessment.

We directly recorded participants’ participation and activity in the program (to assess their engagement) and determined adherence to the intervention by tracking the number of program activities they completed. We also registered the percentage of participants who completed all the activities in the program (ie, completed all nine modules).

### Data Analysis

To detect a reduction in BMI of 1 (SD 1.7), which agrees with the data of a previous study [27], with a two-sided 5% significance level and a power of 80%, and also accounting for an anticipated dropout rate of 10%, a sample size of 52 participants per group was required.

This study used both a between-subjects controlled (analysis at 3 months) and a within-subjects uncontrolled (analysis at 12 months) design; therefore, it involved two different statistical approaches. The between-group comparison (IBI vs WLC) assessed the effect of the IBI on outcomes at 3 months; the within-subject uncontrolled analyses assessed the effect of the IBI over time at the individual level (using pooled data from both groups). This approach was selected because all the WLC participants also subsequently received the IBI to comply with the instructions of the hospital’s human ethics committee. The statistical analysis was performed according to intention-to-treat. Statistical analyses were performed using SPSS version 19.0 for Windows, and the statistical significance was set at *P*<.05 for all our analyses. The data in this study are presented as mean (SD).

#### Analysis at 3 Months

Two-way mixed analysis of covariance (ANCOVA) tests were used to compare the study effects on the BMI, BFM, SBP, DBP, blood glucose and insulin levels, physical activity levels, and functional capacity for aerobic exercise, using time (baseline vs 3 months postintervention) as the within-group factor and group (IBI vs WLC) as the between-group factor. The analysis was adjusted for sex, age, and use of antihypertensive drugs. Effect sizes were estimated using partial eta^2^ and interpreted following the Cohen guidelines [38] for small effect sizes (partial eta^2^=0.01), moderate effect sizes (partial eta^2^=0.06), and large effect sizes (partial eta^2^=0.14).

#### Analysis at 12 Months

After testing the normality (using the Shapiro-Wilk test) of the pooled data from both groups (N=105) at the 12-month follow-up, the following statistical tests were carried out as follows: (1) *t* tests on related samples to compare the baseline vs 12-month follow-up values for the anthropometric variables (ie, BMI and BFM), SBP, DBP, and blood glucose; and (2) Wilcoxon tests to compare the preintervention vs 12-month follow-up values for insulin.

We did not record the patients’ levels of physical activity or their functional capacities for aerobic exercise at 12 months because of the implied complexity of asking participants to record these measurements for 1 year.

Program engagement was analyzed by calculating the percentage of participants who completed the entire program.

## Results

We screened 510 participants in this randomized controlled trial. A total of 405 consecutive subjects were not allocated for randomization, because they declined to participate (n=99) or did not meet the inclusion criteria (n=306): diabetes (106), severe psychiatric disorder (5), taking more than 3 antihypertensive drugs (78), no internet access (87), or other (30). The general characteristics of the study population are shown in Table 1.

**Table 1:** Baseline characteristics of the participants.

Variables	WLC^a^ group (n=50), mean (SD)	IBI^b^ group (n=55), mean (SD)
Age (years)	51.4 (9.3)	54.9 (8.3)
Weight (kg)	81.1 (11.8)	85.4 (12)
Antihypertensive drugs (n)	1.6 (1.1)	2.1 (1.2)
BMI (kg/m^2^)	29.9 (2.6)	30.1 (2.7)
Body fat mass (kg)	27.0 (6.9)	28.2 (6.3)
Systolic blood pressure (mmHg)	128.5 (13.5)	132.2 (14.2)
Diastolic blood pressure (mmHg)	75.8 (9.1)	78.7 (8.1)
Blood glucose (mg/dL)	98 (10)	99 (15)
Insulin (mg/dL)	13.9 (8.7)	15.0 (9.7)
Physical activity counts min^−1^	227 (87)	245 (103)
6-minute walk test (meters)	554 (69)	559 (72)

^a^WLC: wait-list control.

^b^IBI: internet-based intervention.

### Analysis at 3 Months

The results of the 2-way mixed ANCOVA showed a significant decrease in BMI, BFM, and blood glucose after 3 months in the IBI group, with a moderate to large effect size for BMI and BFM; the analysis also highlighted a borderline significant trend (*P*=.05) for DBP and insulin (Table 2). In contrast, we observed a significant increase in BMI and insulin among the WLC group. Additionally, intragroup analysis revealed a statistically significant increase in the functional capacity for aerobic exercise both in the IBI and the WLC groups (Table 2); however, no between-group differences were found. No changes were observed in either group for the level of physical activity measured with accelerometers.

**Table 2:** Intragroup comparisons: baseline vs postintervention (at 3 months)a.

Variables	Wait-list control group (n*=*50)				Internet-based intervention group (n*=*55)		
	Difference (95% CI)	Partial eta^2^	*P* value	Difference (95% CI)		Partial eta^2^	*P* value
BMI (kg/m^2^)	0.3 (0.1-0.5)	.062	.01	–0.4 (–0.7 to –0.2)		.132	<.001
Body fat mass (kg)	0.3 (–0.4 to 1.1)	.018	.34	–1.1 (–1.9 to –0.3)		.125	.009
SBP^b^ (mmHg)	0.0 (–3.7 to 3.7)	.000	.996	–2.6 (–6.1 to 0.9)		.021	.15
DBP^c^ (mmHg)	0.6 (–1.8 to 3.0)	.002	.63	–2.2 (–4.5 to 0.0)		.037	.05
Blood glucose (mg/dL)	2.0 (–1.2 to 5.3)	.015	.22	–3.5 (–6.6 to –0.4)		.048	.03
Insulin (mg/dL)	2.3 (0.4-4.3)	.058	.02	–1.7 (–3.7 to 0.2)		.032	.07
Physical activity counts min^−1^	–8 (–16 to 32)	.005	.52	–6 (–30 to 18)		.002	.64
6MWT^d^ (meters)	21 (8-33)	.108	.001	32 (19-46)		.213	<.001

^a^Difference was calculated as 3 months minus baseline.

^b^SBP: systolic blood pressure.

^c^DBP: diastolic blood pressure.

^d^6MWT: 6-minute walk test.

### Analysis at 12 Months

The results at the 12-month follow-up (Table 3) showed significant improvements in BMI (mean difference [MD] −0.4, 95% CI −0.1 to −0.6; *P*=.005), BFM (MD −2.4, 95% CI −1.1 to −3.6; *P*<.001), DBP (MD −1.8, 95% CI −0.2 to −3.3. *P*=.03), and blood glucose levels (MD −2, 95% CI 0 to −4; *P*=.04) The results of the Wilcoxon tests did not show any changes in insulin values. Regarding engagement in the program, 73.3% (77 out of 105 participants) completed the entire program, comprising of nine modules.

**Table 3:** Comparison of the baseline vs 12-month follow-up values for all participants (N=105).

Variables	Baseline	12-month follow-up	Difference^a^ (95% CI)	*P* value
BMI (kg/m^2^), mean (SD)	30.2 (2.7)	29.8 (2.7)	–0.4 (–0.1 to –0.6)	.005
Body fat mass (kg), mean (SD)	27.6 (6.4)	25.2 (7.9)	–2.4 (–1.1 to –3.6)	<.001
SBP^b^ (mmHg), mean (SD)	130.5 (13.7)	129.3 (14.1)	–1.2 (0.9 to –3.4)	.24
DBP^c^ (mmHg), mean (SD)	77.4 (9.1)	75.6 (9.1)	–1.8 (–0.2 to –3.3)	.03
Blood glucose (mg/dL), mean (SD)	100 (14)	98 (11)	–2 (0 to –4)	.04
Insulin (mg/dL), median (IQR)	16 (12)	15 (9)	–1 (1 to –2)	.64

^a^Difference calculated as 12 months minus baseline.

^b^SBP: systolic blood pressure.

^c^DBP: diastolic blood pressure.

## Discussion

### Principal Results

The present study evaluated the efficacy of a 3-month completely self-administered IBI called *Live better* that aims to promote lifestyle changes in patients who are obese with HTN. Our results showed a significant decrease in the BMI, BFM, and blood glucose levels at 3 months in the IBI group, with a moderate to large effect size for BMI and BFM, and also highlighted a trend toward significance for DBP and insulin levels. Moreover, these improvements in BMI, BFM, and BP were sustained and reached statistical significance for DBP at the 12-month follow-up. When evaluating specific IBIs, several attributes may be associated with increased e-counselling efficacy. These include the duration [22,39], the range of behavior change techniques offered [22,40], the target behaviors to be modified [22], whether specific disease entities are targeted or not [39], and program engagement [35,41].

### Duration

For any intervention to have a significant and sustained effect, a minimum follow-up time of 6 months is required [39]. Indeed, in a recent meta-analysis, Sam Liu et al [22] showed that IBIs lasting at least 6 months were associated with greater BP reductions. These authors argued that the influence of lifestyle interventions on BP may require a critical period for therapeutic changes to appear, and longer interventions might be required to facilitate comprehensive physical changes such as weight reduction. After beginning our study, we found a significant BMI reduction (0.4 kg/m^2^) after 3 months that was sustained after 12 months. Moreover, the mean decrease in BFM (by 1.1 kg) achieved after 3 months had doubled 12 months later (to 2.4 kg). Although this parameter is less frequently used because of its lower feasibility, BFM may be a better marker of cardiovascular risk compared to BMI, which has been criticized because the latter does not always reflect true body fat content [42,43]. Indeed, BMI has some limitations in assessing the risk of obesity-related diseases in subjects with low muscle and high body fat content [44] and in individuals with increased body fat and a normal BMI.

Consistent with our results, a Cochrane meta-analysis concluded that interactive computer-based interventions, compared to no intervention or minimal interventions (eg, pamphlets or usual care), are an effective tool for enhancing weight loss and weight maintenance [12]. In addition, providing support oriented towards self-management while patients change their lifestyle leads to improved health outcomes [24,45] and better long-term effects [46].

Concerning BP values, we found a nonsignificant decrease in SBP and DBP after 3 months, which was sustained and even reached statistical significance after 12 months in the case of DBP (−1.8 mmHg). These results are slightly lower than those reported in the Internet Lifestyle Counselling meta-analysis (−3.8 mmHg for SBP and −2.1 mmHg for DBP) by Liu et al [22] and are also lower than the BP reductions reported by other meta-analyses that considered face-to-face lifestyle counselling [9,47]. The lack of a stronger significant effect in our study may be because the BP at baseline was already well controlled by antihypertensive medications [48-50] by the specialized HTN unit. Effectively, starting from normal mean values in treated hypertensives, even greater weight losses would probably not have induced further reductions of BP. This phenomenon is known as the “floor effect” and must be accounted for [51].

### Range of Behavior Change Techniques

Two recent meta-analyses that evaluated the BP lowering [22] and weight loss [40] achieved by e-counselling lifestyle interventions reported that their efficacy depended on the number of intervention components they included. The meta-analysis by Liu et al [22] found that BP was preferentially, and significantly, reduced by interventions providing a wider range of behavioral change techniques and suggested that “a critical number of techniques (at least 5) may be required to build a flexible repertoire of skills that are necessary to overcome situational stressors that might otherwise impede therapeutic lifestyle change”. These authors reported that the behavioral change techniques present in more than 50% of the successful IBIs shared the following features: provisioning general information about the consequences of the patient’s behavior (86% of studies), incorporating feedback on performance (86%), prompting behavioral self-monitoring (71%), and giving instructions on how to perform targeted behavioral changes (71%). Likewise, Khaylis et al [52] also conducted a systematic review of efficacious technology-based weight loss interventions and identified several similar components (self-monitoring, counsellor feedback and communication, social support, structured programs, and individually tailored programs) that were key to their success. Other effective components identified in the technology-based weight loss literature as potential factors that increase intervention effectiveness are goal setting, motivational interviewing, and incentives [30,53,54].

All the behavioral change techniques mentioned by Liu et al [22] and Khaylis et al [52] were present in our intervention, except for feedback incorporation, motivational interviewing, and social support, as these were not possible given the self-administered nature of our intervention, which maintained its low monetary and time costs. Moreover, this trial was distinct insofar as it also incorporated problem-solving techniques focused on the regulation of emotional eating (module 5) and the difficulties associated with body image and assertiveness (module 7). As Katan [55] suggested, cognitions and feelings have a big impact on behavior during dieting and, thus, may strengthen or disrupt treatment engagement and compliance with clinical prescriptions. Indeed, psychological factors and processes mediate every behavior change and differently affect both the initiation and maintenance phases of change [56].

### Target Behaviors to Be Modified

Exercise and diet are two major target behaviors for modification by internet-based e-counselling interventions designed to prevent or treat cardiovascular disease risk factors such as obesity and HTN. Numerous studies suggest that automated, self-guided, internet-based lifestyle counselling (e-counselling) programs can evoke meaningful improvements in daily physical activity [57,58] and dietary behaviors [58,59]. Moreover, meta-analysis reviews indicate that exercise and diet, provided by conventional programs or by IBIs, significantly decrease cardiovascular risk factors [9,16,47,60,61]. Our *Live better* intervention focused on modifying both of these target behaviors, because it has been shown that tackling them simultaneously is more effective at promoting weight loss than targeting either alone [62].

After 3 months, our results showed improvements in the patients’ functional capacities for aerobic exercise with a large effect size in both sexes (partial eta^2^=0.213) in the IBI group. Nevertheless, caution must be taken in interpreting these results, as this variable also increased with a moderate effect size in the WLC group (partial eta^2^=0.108). It is worth considering that a learning effect over a 2-month period has been documented with repeated administration of the 6MWT [63]. In contrast to subjective physical activity estimations such as questionnaires, in our study we used an accelerometer to obtain precise and objective measurements of activity levels in individuals who are overweight or obese [64]. We did not find significant intergroup or intragroup differences after 3 months. However, these results should be interpreted with caution since the accelerometers do not accurately quantify activities such as resistance training or swimming (included in the recommendations of the IBI). The fact that the reduction in BFM doubled in the time between the end of the intervention (3 months) and the end of the study (12 months), while simultaneously maintaining a constant BMI, may indicate that the patients had increased muscle mass secondary to higher levels of physical activity during this period. Thus, future studies will be required to measure muscle mass to test this hypothesis.

### Targeting Specific Disease Entities

*Live better* was specifically designed to treat HTN in patients who are overweight or those with type I obesity, and its implementation resulted in modest but positive measurable results in body composition and BP. In general, internet-based programs are more successful if they are targeted at specific disease entities such as HTN [39]. Although our study listed diabetes as an exclusion criterion, and our intervention did not specifically focus on this disease, improved glucose metabolism was also relevant in our study because we detected a significant and sustained decrease in glucose levels among our patients. The close relationship between glucose metabolism variables and healthy living through physical activity and healthy eating is well known. In line with this, a recent high-quality randomized lifestyle-intervention trial conducted over 12 and 24 months in individuals with type 2 diabetes mellitus or impaired fasting glucose levels showed the IBI to be useful in reducing fasting plasma glucose [21].

### Program Engagement

In terms of program engagement, the percentage of participants who completed our entire program was high (77/105, 73.3%) and was similar to the levels reported in related eHealth interventions [35,65]. Increasing knowledge about healthy lifestyles and making tailor-made prevention programs possible can empower individuals and improve their adherence to interventions [66]. In this sense, the wide and still-growing access and use of the internet has become a major resource in the assessment of health information [67]. This is especially true for adults with chronic conditions who are more likely to seek health information on the internet than their counterparts with acute ailments [68-70]. Using modern information and communication technologies to deliver physical activity and diet interventions is particularly promising considering the increased proliferation of such technologies in many developing countries.

The internet is an efficient way to prevent and treat chronic conditions by promoting healthy lifestyles because it can reach more individuals (including those with limited access to health services or with low levels of social support) and can provide more intensive contact at potentially lower costs than conventional face-to-face programs [71-74]; it can provide immediate, easy access, individually tailored (one-to-one), and “permanent” (accessible at any time) behavioral-change support by delivering care to patients in the comfort of their own homes with self-paced delivery [35,40,73]; and it is less intrusive than traditional methods and can more easily be implemented in an environment that is less threatening than a hospital [10].

However, despite the encouraging results of this study and other studies on internet-based programs, the goal of web-based interventions is not to replace in-person care but rather to maximize care. Of note, internet interventions also have potential disadvantages such as their inability to recognize comorbidities that would not have become apparent without the patient implementing lifestyle changes. Furthermore, internet use depends on age, income, education level, and digital skills, and there may be participation bias and lower response rates because of technical problems or different levels of computer experience among participants [5]. In addition, ironically, internet use is a sedentary behavior, which is known to be an independent risk factor for cardiovascular disease, so caution should be exercised when designing these programs so that they prevent, rather than encourage, further sedentary attitudes and behaviors.

### Limitations

The main limitation of this study was its lack of a control group for the analysis carried out at 12 months. Although the positive results at 3 months remained at the 12-month follow-up visit, the follow-up analyses were based on uncontrolled data and, thus, should be interpreted with caution. Controls eliminate possible alternate explanations of experimental results, especially those of confounding variables and experimental bias, which allows investigators to control for threats to validity.

Furthermore, the participants we enrolled had demonstrated an initial level of motivation to engage in an eHealth program. Therefore, our findings may only be generalizable to individuals with internet access who are similarly interested in such eHealth interventions [67]. In addition, our participants were recruited from a public hospital (rather than a private one), which may have influenced our results because sociodemographic status (which could correlate with the use of public hospitals) has been related to treatment adherence for chronic conditions [75,76]. Another possible limitation of the study was the inclusion of BMI as the primary outcome. Although the waist circumference or waist-hip ratio are considered better anthropometric parameters to reflect the risk of cardiovascular disease associated with obesity, the BMI clinical tool has been shown to have the least bias during assessment [73]. Furthermore, BMI measurements in our study were complemented with segmental body-fat distribution analysis measured with bioelectrical impedance. Even though intervention acceptability is related to its eventual effectiveness [77], we did not assess this data in our study, and thus, we do not know how well the participants accepted this IBI. Finally, we did not measure physical activity levels or functional capacity for aerobic exercise at the 12-month follow-up.

### Conclusions

In summary, we explored the effects of implementing a 3 month, self-administered IBI involving different learning techniques and aimed at promoting lifestyle changes (physical activity and healthy eating) in patients who are obese with HTN. Overall, the participant engagement was high, and we observed moderate to large effect sizes in relation to BMI and BFM reductions. In addition, there was a favorable trend towards a relation to BP, which reached statistical significance at the 12-month follow-up. Taken together, these findings suggest that our tailored approach for delivering a lifestyle change intervention to patients who are obese with HTN provides positive health benefits. Simple strategies that can easily be incorporated into daily living in a scalable and cost-effective way can empower patients by educating them about health, thus, increasing their confidence and promoting self-management. Future research should investigate the acceptability and cost-effectiveness of self-administered internet-based interventions in populations with different sociodemographic status.

## Appendix

Multimedia Appendix 1 Topics and objectives covered in the modules.

Multimedia Appendix 2 CONSORT e-HEALTH checklist (V. 1.6.1).

## Abbreviations

ANCOVA: analysis of covariance
BFM: body fat mass
BP: blood pressure
D: diastolic
eHealth: electronic health
HTN: hypertension
IBI: internet-based intervention
MD: mean difference
S: systolic
WLC: wait-list control
6MWT: 6-minute walk test.
