# Supplementary material for: Impact of a Web-Based Exercise and Nutritional Education Intervention in Patients Who Are Obese With Hypertension: Randomized Wait-List Controlled Trial
Source: J Med Internet Res. 2020 Apr 14;22(4):e14196. doi: 10.2196/14196 (PMC7189251; doi:10.2196/14196)
Supplement: Multimedia Appendix 1 [file jmir_v22i4e14196_app1.docx]

Multimedia Appendix. Topics and objectives covered in the modules

| **Module and topics** | **Objectives** | **Screenshots of the intervention and self-monitor tools** |
| --- | --- | --- |
| M 0 - Welcome | - To welcome to the program; - To start developing motivation to change; - To explain what is and how it will be presented the program with the 9 modules; - To explain which is an Online Program; - To present main points that will be needed by the user to get succeed. | 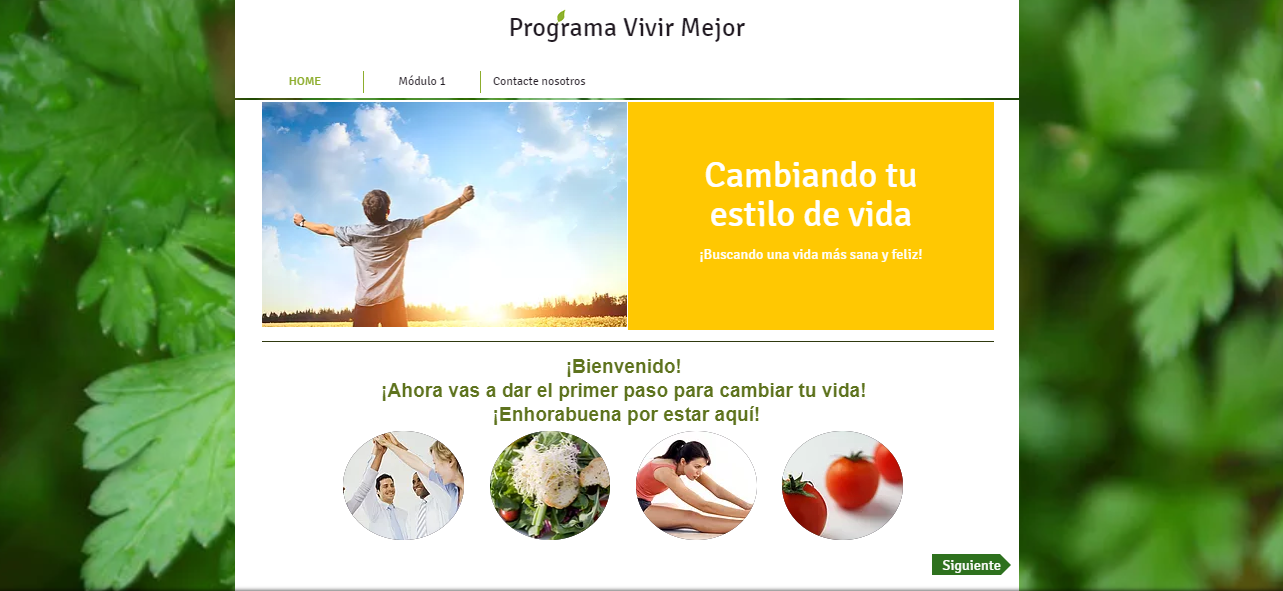  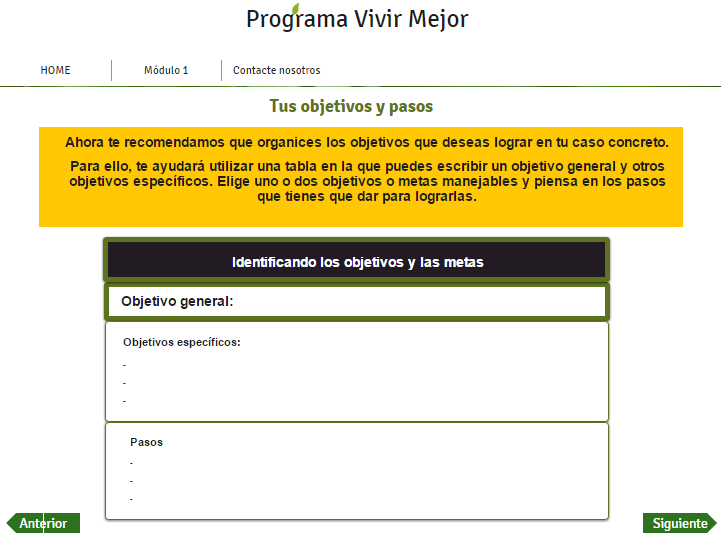  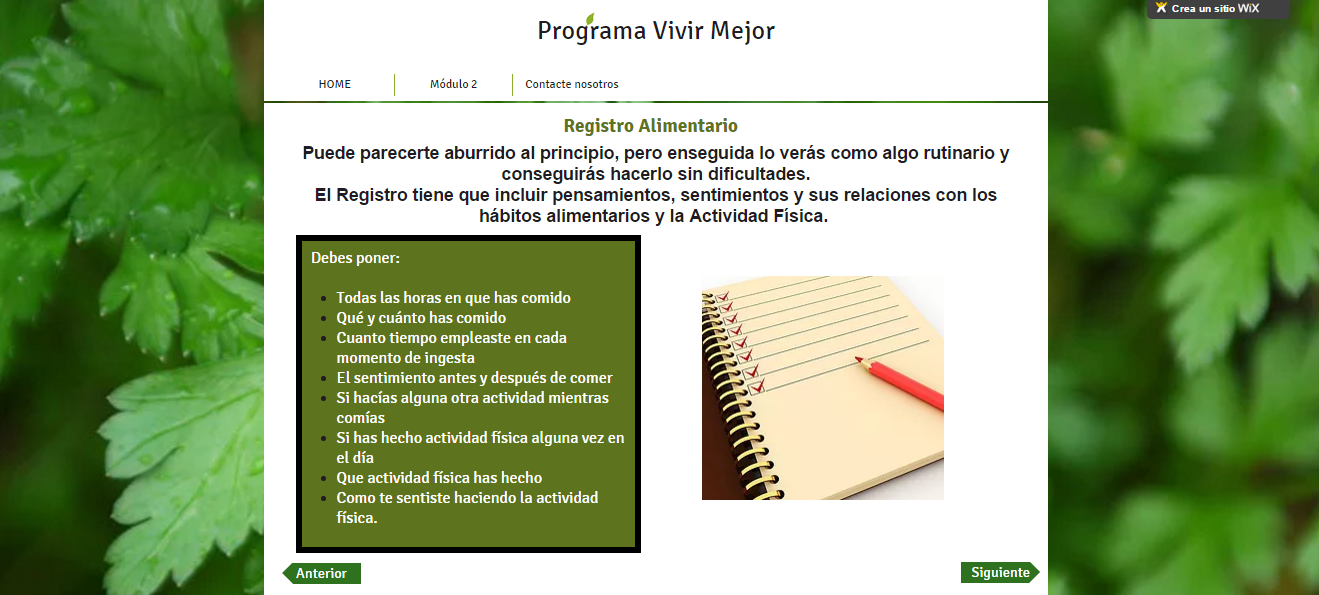  *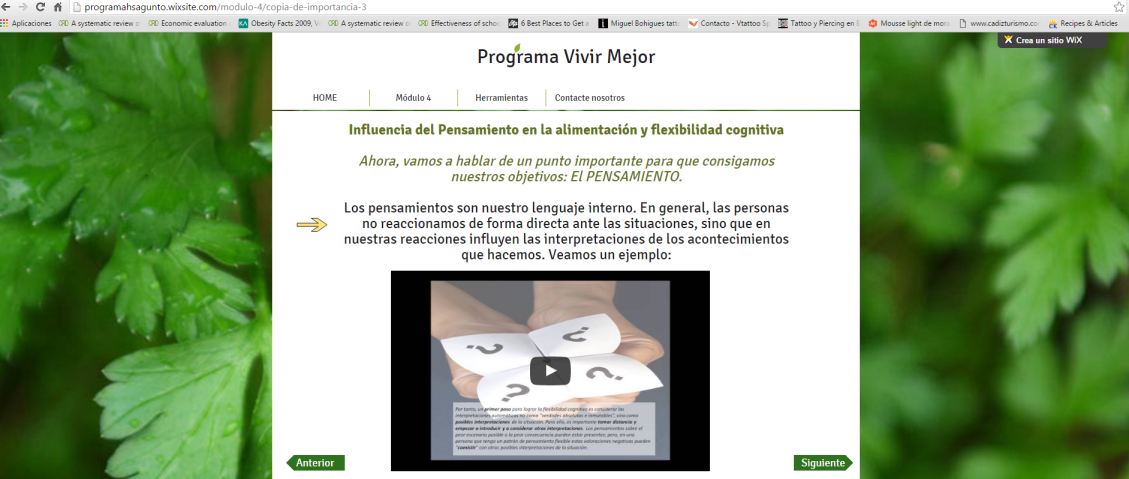*  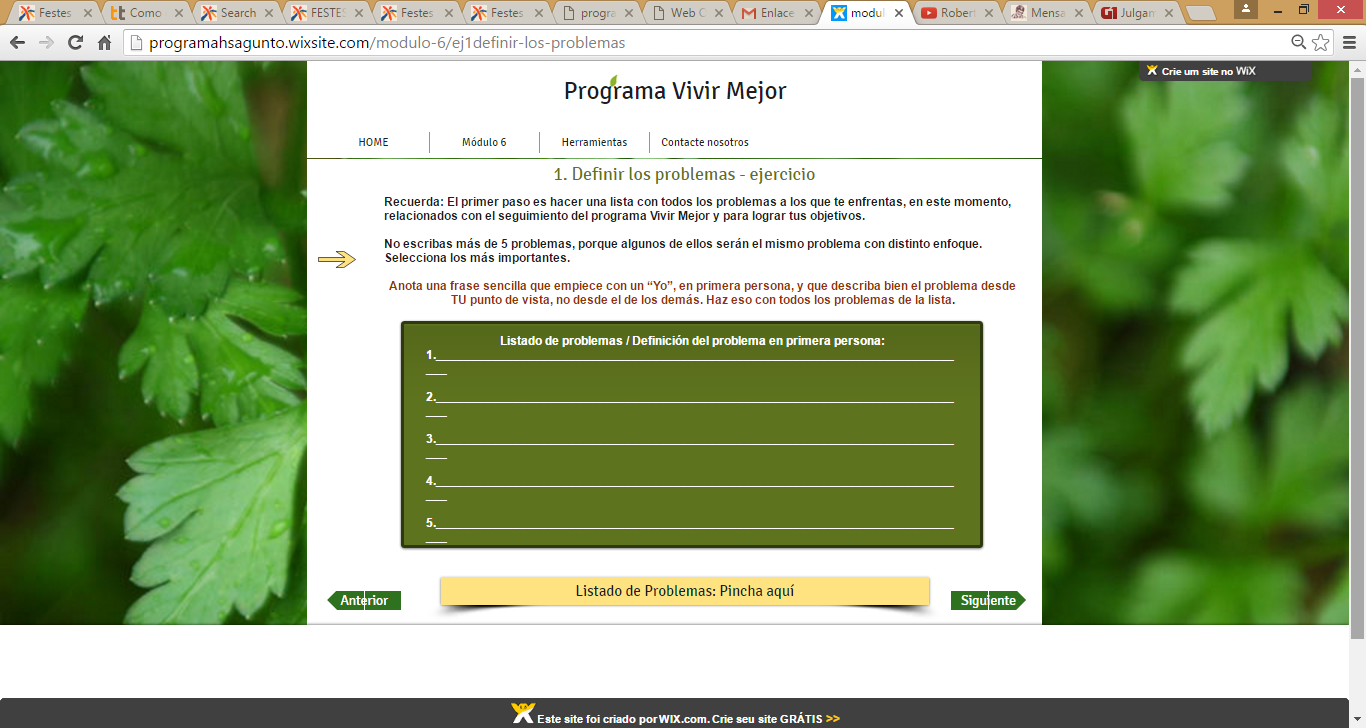  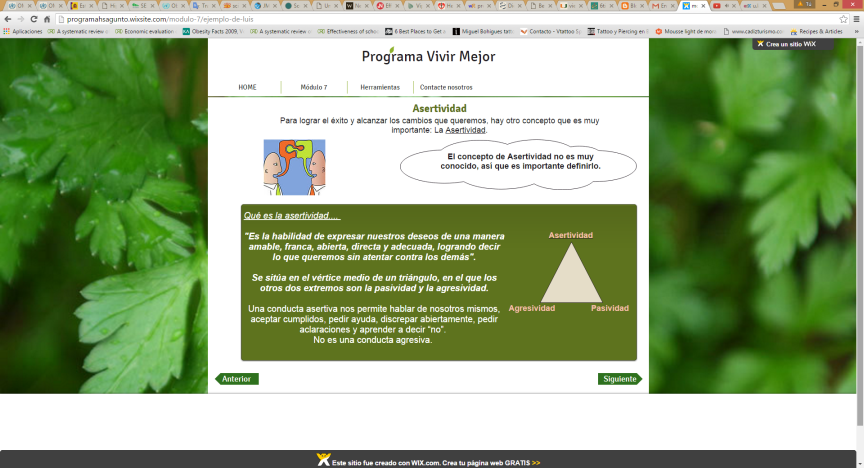  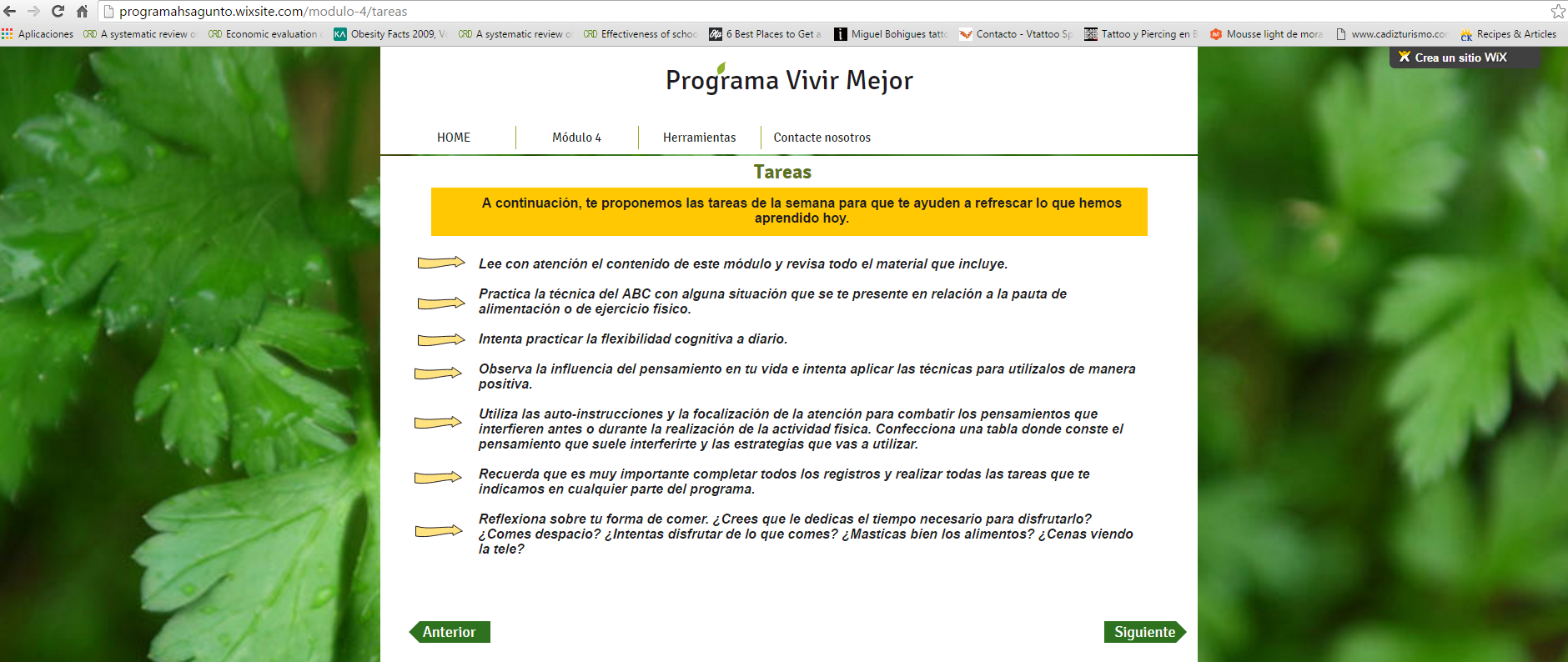  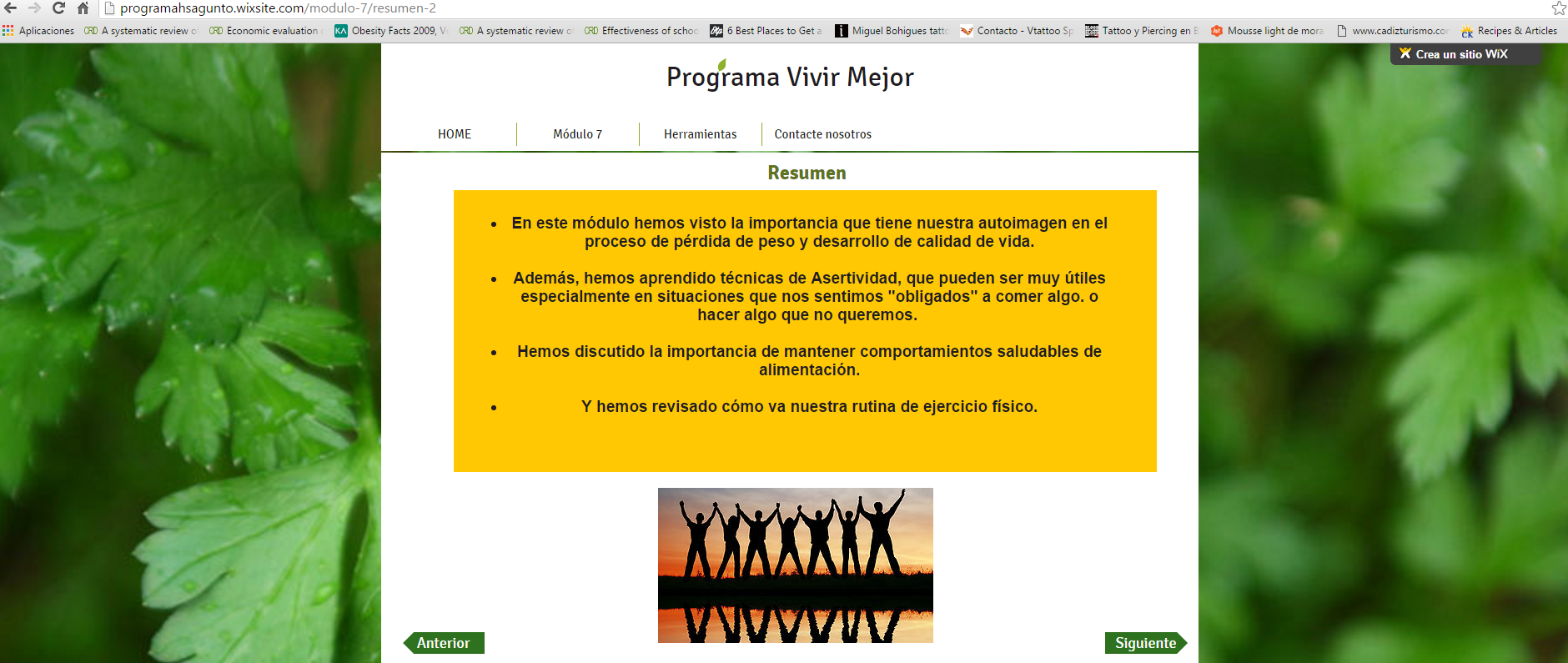 |
| M 1 – Motivation for change | - To know the importance of motivation to change lifestyle; - To know the costs and benefits of maintaining certain habits and behaviors and the costs and benefits of change; - To decide what relevant aspects of your day would be advisable for you to change; - To learn to set specific and manageable goals to achieve the changes proposed. |  |
| M 2 - **Nutrition education and education on physical activity and exercise** | - To reflect on why eating well; - To have general information about the composition of foods; - To learn what physical activity is and difference it from exercise or sport; - To understand the role of physical activity in changing lifestyle and weight loss; - To provide the keys to start being more active in everyday life; - To know what a pedometer is and what it does. |  |
| M 3 - **Barriers to change habits, healthy eating and how to be active.** | - To identify possible barriers to healthy eating and physical activity, preparing to face them and overcome it; - To discuss the importance of meals: Breakfast; to learn the difference between different types of exercise; - To learn what type of exercise is best recommended; - To know some general basic recommendations for physical activity. |  |
| M 4 - **Modification of irrational beliefs and influence of thoughts.** | - To know factors related to maintaining an unhealthy lifestyle; - To identify the role of thinking in the choices and actions about food and physical activity; - To develop self-knowledge strategies, by the technique of ABC; - To learn little tricks that will help to eat more efficiently; - To discuss the importance of meals: lunch and snack. |  |
| M 5 - **Emotional regulation, emotional eating and self-control** | - To identify what it is and when it happens emotional eating; - To discover how to cope with emotional eating; - To work on strategies to implement self-control in situations where a compulsive eating behavior can be observed; - To develop strategies of emotional regulation; - To suggest tools to optimally handle specific situations of emotional eating. |  |
| M 6 - ***Strategies for overcoming obstacles and solving problems*** | - To identify obstacles and barriers that often appear in the process of lifestyle changing; - To provide the process of taken awareness of the problem related to lifestyle change (change the food and PA pattern); - to provide learning other coping strategies: the problem solving technique. |  |
| M 7 -**Intervention on body image difficulties and Assertiveness** | - To work or personal image one has of the body; - To identify concerns with body image; - To encourage the development of a positive body image; - To promote a better understood about assertiveness: what is, how to use it, and interesting techniques. |  |
| M 8 - **Relapse Prevention** | - To fix the foundation for achievement, relative to healthy food regimen, activity and exercise, and the new lifestyle takes hold; to strengthen the changes obtained; - To set strategies to keep the changes obtained; - To prevent possible relapse. |  |
